# Spectroscopic data for the G-quadruplex DNA to duplex DNA reaction

**DOI:** 10.1016/j.dib.2015.10.039

**Published:** 2015-11-05

**Authors:** Oscar Mendoza, Juan Elezgaray, Jean-Louis Mergny

**Affiliations:** a University of Bordeaux, 33600 Bordeaux, France; b INSERM, ARNA Laboratory, U869, IECB, F-33600 Pessac, France; c CBMN, CNRS UMR-5248, F-33600 Pessac, France

## Abstract

This article describes additional data related to a research article entitled “Kinetics of Quadruplex to Duplex Conversion” (Mendoza et al. 2015 [1]). We followed the opening reaction of a series of intramolecular G-quadruplex structures by the addition of their corresponding complementary strand. Fluorolabeled complementary strands allowed to monitor the reaction in real-time. An adapted kinetic model was then applied in order to obtain the kinetic parameters of this reaction.

We present a series of kinetic traces providing raw data of the G4 opening reaction and the fitting model applied in every case. In addition CD spectra and UV melting data is also provided to confirm the stability of all the DNA structures considered (G-quadruplex and duplex DNA).

**Specifications table**

**Table:** 

Subject area	*Biophysics*
More specific subject area	*Nucleic acids*
Type of data	*Tables and figures*
How data was acquired	*JASCO CDF-426S, SAFAS spectrophotometer, Tecan Infinite M1000 PRO*
Data format	*Raw data emission intensity and CD spectra; normalized UV-melting profiles*
Experimental factors	*Quadruplex DNA systems were annealed at* 1 μM
Experimental features	*Spectroscopic characterization of G-quadruplex structures and their unfolding process.*
Data source location	*Bordeaux (France)*
Data accessibility	*Data within this article*

**Value of the data**

• Individual fitting models can be used to obtain kinetic constants of G-quadruplex unfolding reactions.
• The DNA sequences considered in this study fold into well stablished G-quadruplex structures.
• The stability of the G-quadruplex and the polarity of the strand were characterized by spectroscopic studies (CD and UV).

## 1 Data

The unfolding reaction of a series of G-quadruplex structures by addition of their complementary strand was monitored by fluorescence techniques [1]. Kinetic constant of this G4 opening process was obtained by playing individual fitting models to the fluorescence data. Furthermore, DNA structures considered in this studied (G-quadruplexes and duplex DNA) were characterized spectroscopically. Their thermal stability (Tm) was studied by monitoring the thermal melting process of the DNA motif by UV (at 260 nm and 295 nm for duplex DNA and G4 DNA respectively). In addition, the polarity of the G-quadruplex was also studied by circular dichroism, which confirmed the correct folding of the G4 structures.

**Fig. 1:**
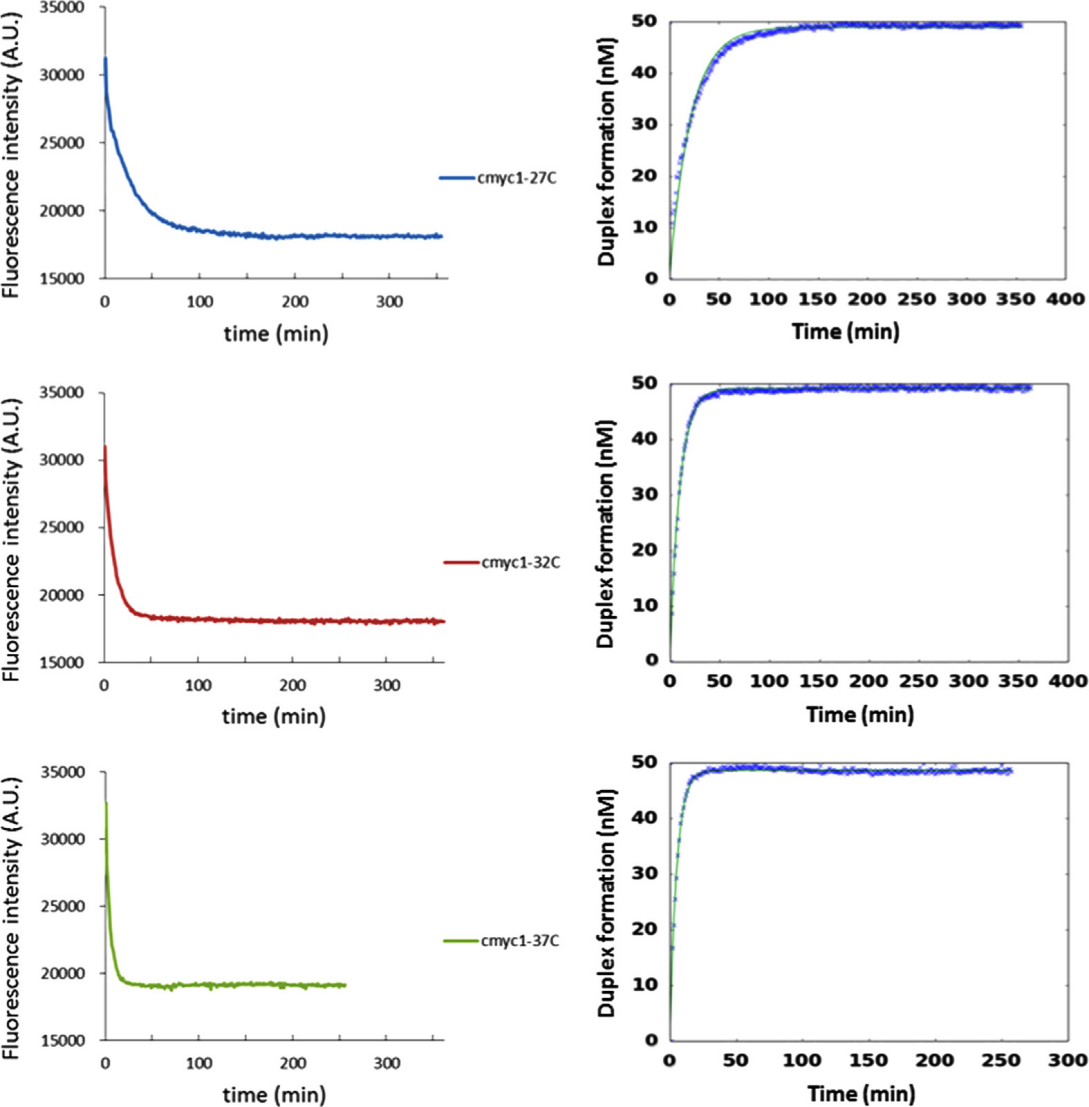
Raw data emission intensity (left) and normalized fitting model (right) upon duplex DNA formation for **cmyc1** system at 1 mM KCl and 27 °C, 32 °C and 37 °C.

**Fig. 2:**
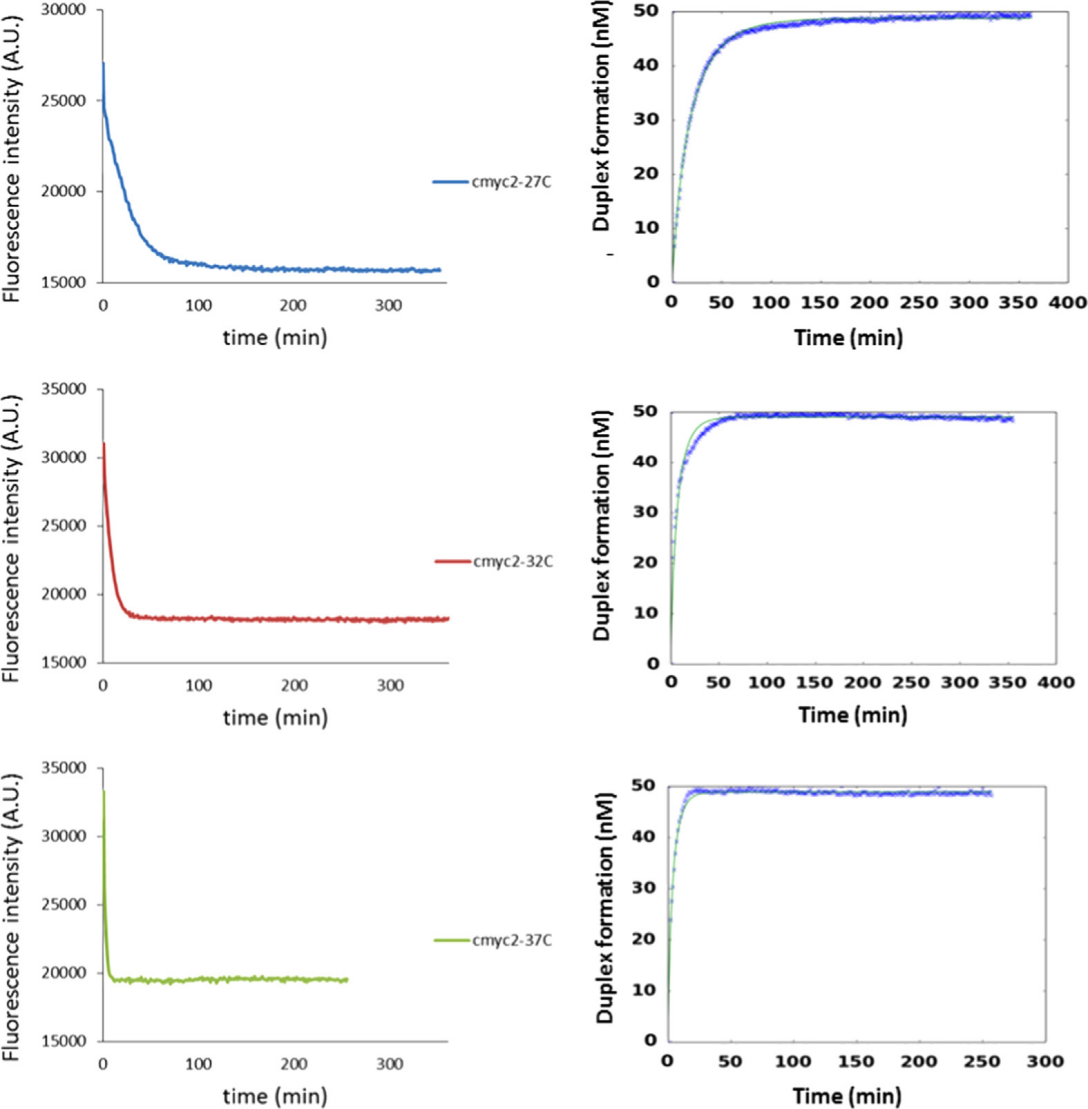
Raw data emission intensity (left) and normalized fitting model (right) upon duplex DNA formation for **cmyc2** system at 1 mM KCl and 27 °C, 32 °C and 37 °C.

The data provide in Fig. 1, Fig. 2 shows the raw emission data for the fluorescence intensity of the labeled DNA strands. When the reaction takes places, the formation of the stable duplex quenches the emission intensity. Fig. 1, Fig. 2 show also the kinetic fitting model applied to obtain the corresponding kinetic constant of every opening reaction.

**Fig. 3:**
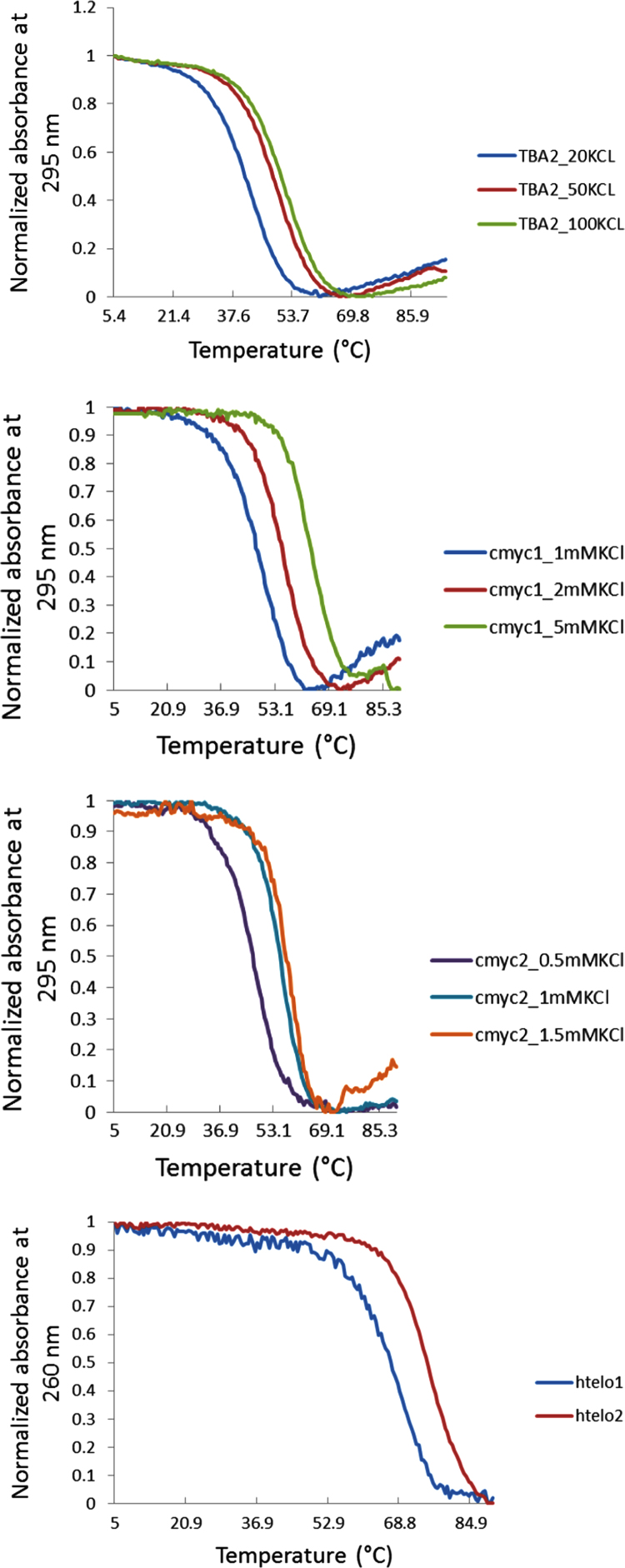
Melting profiles (normalized absorbance at 295 nm) obtained for system **TBA2** (at 20, 50 and 100 mM KCl), **cmyc1** (at 1, 2 and 5 mM KCl), **cmyc2** (at 0.5, 1 and 1.5 mM KCl), **htelo1** (100 mM KCl) and **htelo2** (100 mM KCl).

**Fig. 4:**
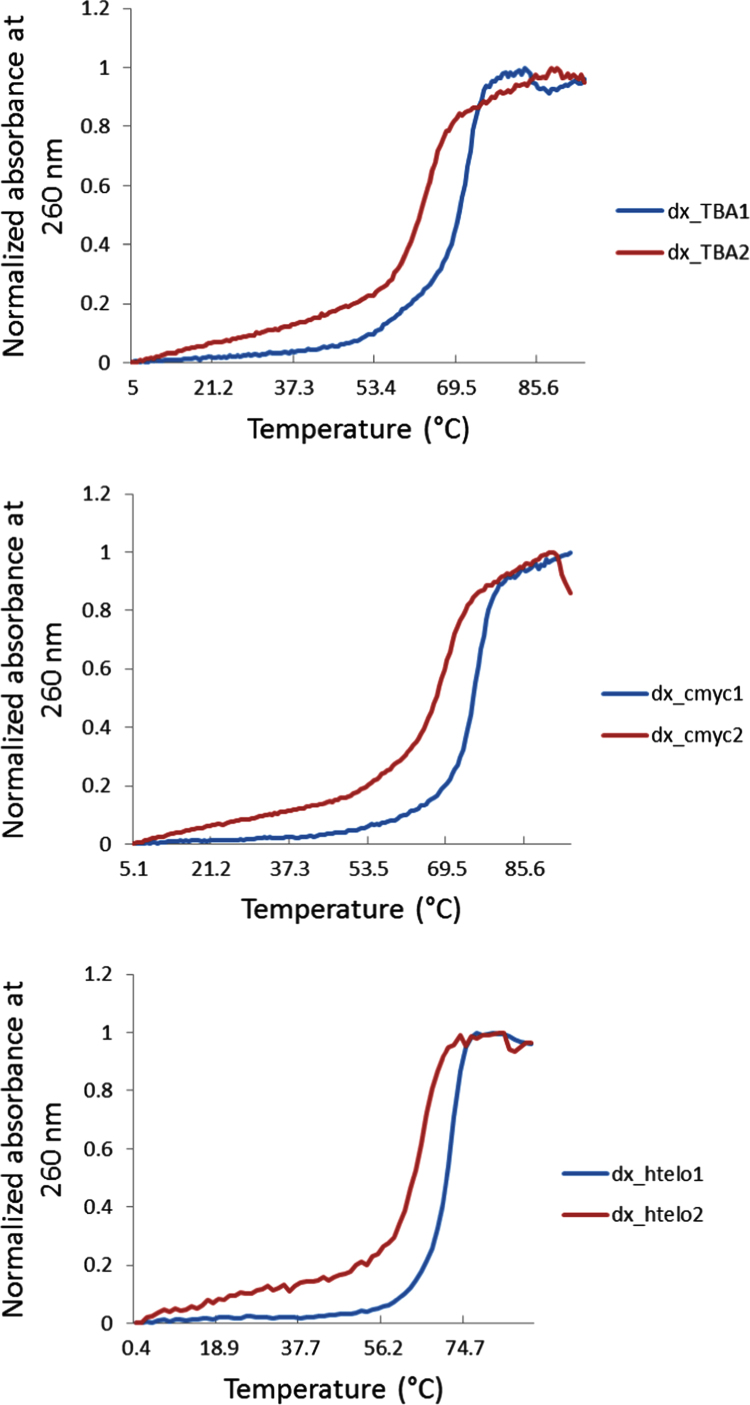
Melting profiles (normalized absorbance at 260 nm) obtained for 4 μM DNA duplex: **dx_TBA2**, **dx_TBA2**, **dx_cmyc1**, **dx_cmyc2**, **dx_htelo1** and **dx_htelo2**.

The thermal stability of all DNA structures considered in this research is also provided. The Tm of the G-quadruplex motifs **TBA2**, **cmyc1**, **cmyc2**, **htelo1** and **htelo2** and the duplex structures **dx_TBA2**, **dx_TBA2**, **dx_cmyc1**, **dx_cmyc2**, **dx_htelo1** and **dx_htelo2** was calculated. Fig. 3, Fig. 4 show the thermal melting profiles for the G-quadruplex and duplex structures respectively.

**Fig. 5:**
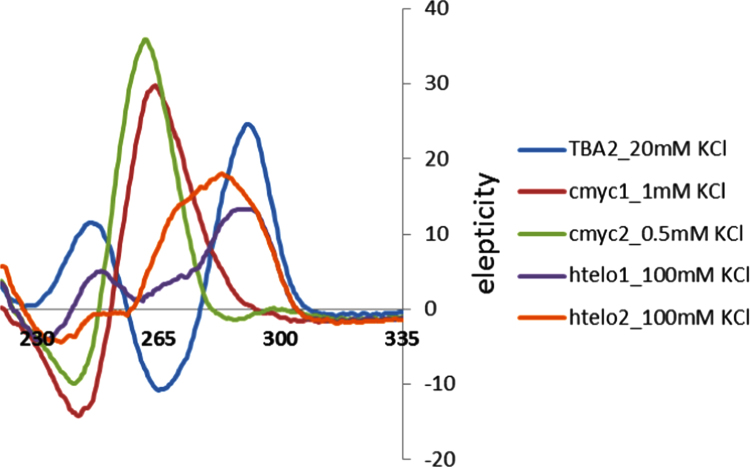
CD spectra in 10 mM cacodylate buffer (pH 7.2) of the previously annealed substrate **TBA2** (containing 20 mM KCl), **cmyc1** (containing 1 mM KCl), **cmyc2** (containing 0.5 mM KCl), **htelo1** (containing 100 mM KCl) and **htelo2** (containing 100 mM KCl).

**Fig. 6:**
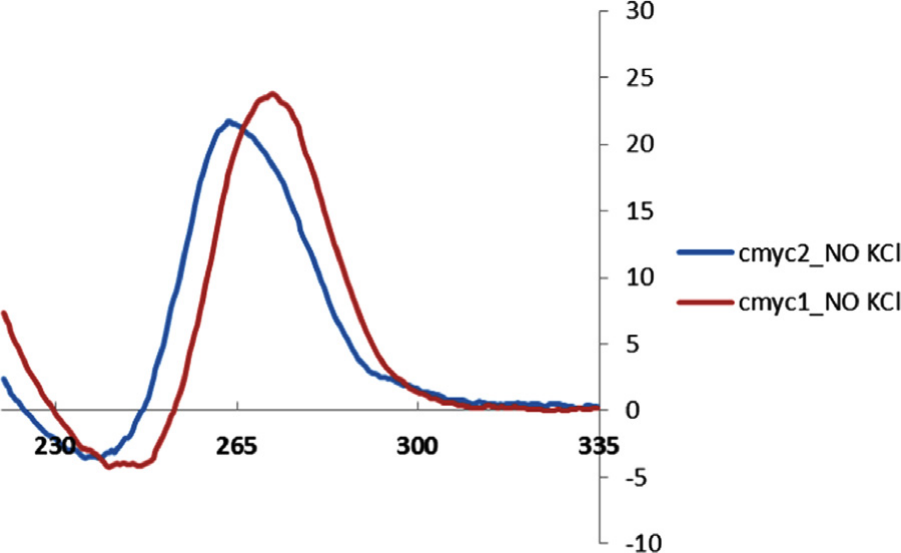
CD spectra in 10 mM cacodylate buffer (pH 7.2) **cmyc1** and **cmyc2** containing 100 mM LiCl and no KCl.

Circular dichroism was used to confirm the presence of a G4 motif in **TBA2**, **cmyc1**, **cmyc2**, **htelo1** and **htelo2** at the lowest KCl concentration considered in this study (thus 20 mM KCl, 1 mM KCl, 0.5 mM KCl, 100 mM KCl and 100 mM KCl respectively). Fig. 5, Fig. 6 show that in all the cases, a typical signature of a quadruplex motif was found. Finally Table 1 shows the calculated kinetic constants for the opening reaction of the quadruplex motif **htelo1**.

## 2 Experimental design, materials and methods

*Kinetic Fitting:* we used a fitting procedure that minimizes the error between the experimental data (concentration of the final double stranded complex as a function of time) and the solution of the model as obtained with an ordinary differential equation (ODE) integrator (Odeint from the Scipy library) [2].

*UV-Thermal Melting Curves:* were performed in 10 mM cacodylate buffer (pH 7.2) and 100 mM mixture of LiCl+KCl for quadruplex systems and 100 mM LiCl for duplex structures. DNA systems were prepared at 4 μM in the corresponding buffer and then denatured at 90 °C for 5 min and cooled down rapidly in ice. Thermal denaturation curves were obtained with a SAFAS spectrophotometer using quartz optical cells of 1 cm pathlength using a scan rate of 0.3 °C per min and following the variation of UV absorption at 295 nm and 260 nm.

*Circular dichroism (CD) spectroscopy**:*** CD experiments were performed with a JASCO J-815 spectropolarimeter equipped with a JASCO CDF-426S Peltier temperature controller, using quartz cells of 1 cm path length. The scans were recorded from 210 to 335 nm wavelength with the following parameters: 0.5 nm data pitch, 2 nm bandwidth, 100 nm min^−1^ scanning speed, and are the result of 3 accumulations.

## Figures and Tables

**Table 1:** Calculated reaction rate constants of the unfolding reaction of **htelo1** at 100 mM KCl concentrations and several temperatures. Errors calculated from the average of three independent repetitions.

Temp. (°C)	Reaction rate constant K_2_ (s^−1^)
27	(2.18±0.18)×10^−3^
32	(2.28±0.37)×10^−3^
37	(3.19±0.41)×10^−3^
42	(4.51±0.41)×10^−3^
